# Immune/inflammatory parameters as potential predictors of high-lethality suicidal behavior in individuals with/without psychiatric conditions: a retrospective single-center study

**DOI:** 10.1192/j.eurpsy.2025.759

**Published:** 2025-08-26

**Authors:** L. M. Martorell Mensua, W. G. Vidal Cachay, M. Quesada Franco, A. Beneria Gonzalez, J. A. Ramos Quiroga, A. Motger Albertí, G. Arteaga-Henriquez, D. Braquehais Conesa

**Affiliations:** 1 Psychiatry, Vall Hebron Hospital; 2 Psychiatry; 3 Mental health and addictions, VHIR; 4 Psychiatry and Legal Medicine, Universitat Autonoma de Barcelona (UAB); 5 School of Medicine, Universitat Internacional de Catalunya, Barcelona, Spain

## Abstract

**Introduction:**

Accumulating research has suggested a possible role of the immune/inflammatory response system in the pathophysiology of suicidal behavior, more specifically of specifically high-lethality suicide attempts (SA). To count with reliable and affordable biological markers of high-lethality SA to complement clinical assessment for early detection of individuals at a high risk of committing suicide is thus mandatory.

**Objectives:**

To assess if immune/inflammatory parameters may differ between suicide attempters with and without SA, taking into account the type of suicide method. The odds of repeating a high-lethality SA in the future will also be explored.

**Methods:**

In this retrospective observational single center study, medical records of suicide attempters admitted to the Emergency Department at Vall d’Hebron University Hospital (Barcelona, Spain) between 2017-2021, will be reviewed. The following immune/inflammatory parameters (i.e., total and differential white blood cell count, platelet counts, C-reactive protein levels) will be extracted for comparisons between subjects without a history of previous suicide attempt (SA), and those with a history of previous SA. Additionally, the following ratios/indexes will be calculated as a proxy of subject’s inflammatory status: neutrophil-to-lymphocyte ratio (NLR), basophil-to-lymphocyte-ratio (BLR), monocyte-to-lymphocyte ratio (MLR), platelet-to-lymphocyte ratio (PLR), systemic inflammatory response index (SIRI). Analyses will be controlled for clinical and sociodemographic variables, such as age, gender and/or primary psychiatric diagnosis. Analyses will be also stratified according to the attempt method. Moreover, the capability of the previously mentioned parameters to predict a high lethality SA or to commit suicide in the coming two years will also be evaluated.

**Results:**

Results from the interim analysis will be presented at the congress.

**Conclusions:**

Peripheral immune/inflammatory parameters may allow us to discriminate subjects at risk of committing suicide. In case of positive findings, immune/inflammatory parameters could be incorporated in the comprehensive evaluation of high-lethality SA in individuals admitted to the emergency setting, contributing to improve early detection of suicide risk.

**Disclosure of Interest:**

L. Martorell Mensua Shareolder of: First authorship, W. Vidal Cachay Shareolder of: First authorship, M. Quesada Franco: None Declared, A. Beneria Gonzalez: None Declared, J. Ramos Quiroga: None Declared, A. Motger Albertí: None Declared, G. Arteaga-Henriquez Shareolder of: Last authorship, D. Braquehais Conesa Shareolder of: Last authorship.

